# Supplementary material for: MetaRibo-Seq measures translation in microbiomes
Source: Nat Commun. 2020 Jun 29;11:3268. doi: 10.1038/s41467-020-17081-z (PMC7324362; doi:10.1038/s41467-020-17081-z)
Supplement: Supplementary file 10 — Supplementary Data 7 [file 41467_2020_17081_MOESM10_ESM.zip › File2/Confidence_High_Taxonomy/131817_out.krona.html]

Javascript must be enabled to view this page.

members
magnitude
magnitudeUnassigned
count
unassigned
taxon
rank

131817\_out

179

179
2
superkingdom

1
phylum
1239

186801
class
1

186802
order
1

family
186803
1

genus
841
1


SRS148159\_contig\_number\_16876
166486
species
1

178
phylum
976

178
class
200643

order
171549
178

family
1853231
177

177
genus
283168
33

SRS011239\_contig\_number\_contig-100\_747.134305SRS011405\_contig\_number\_10015SRS013521\_contig\_number\_12514SRS014412\_contig\_number\_12871SRS014923\_contig\_number\_23141SRS015694\_contig\_number\_contig-100\_559.560SRS015890\_contig\_number\_22664SRS019787\_contig\_number\_3373SRS020233\_contig\_number\_53818SRS023583\_contig\_number\_10035SRS023715\_contig\_number\_23764SRS024549\_contig\_number\_2866SRS045244\_contig\_number\_4759SRS048060\_contig\_number\_834SRS049712\_contig\_number\_14636SRS050299\_contig\_number\_contig-100\_3286.33760SRS050941\_contig\_number\_3517SRS050998\_contig\_number\_contig-100\_1586.81365SRS064276\_contig\_number\_29474SRS064645\_contig\_number\_contig-100\_2677.2677SRS075821\_contig\_number\_917SRS076756\_contig\_number\_19368SRS077335\_contig\_number\_5195SRS077392\_contig\_number\_contig-100\_841.157816SRS1041039\_contig\_number\_5309SRS142923\_contig\_number\_672SRS143181\_contig\_number\_3785SRS143523\_contig\_number\_4569SRS143895\_contig\_number\_12801SRS145497\_contig\_number\_contig-100\_1183.88691SRS147425\_contig\_number\_3496SRS148874\_contig\_number\_contig-100\_1454.81003SRS149244\_contig\_number\_contig-100\_2313.41611


SRS011084\_contig\_number\_18802SRS011134\_contig\_number\_25296SRS012849\_contig\_number\_31496SRS012969\_contig\_number\_17440SRS013098\_contig\_number\_contig-100\_248.233363SRS013800\_contig\_number\_26077SRS014683\_contig\_number\_13705SRS015264\_contig\_number\_665SRS015431\_contig\_number\_44835SRS015663\_contig\_number\_19202SRS015782\_contig\_number\_27007SRS015960\_contig\_number\_17587SRS016018\_contig\_number\_5914SRS016056\_contig\_number\_1188SRS016095\_contig\_number\_26117SRS016335\_contig\_number\_42407SRS016954\_contig\_number\_3820SRS017191\_contig\_number\_5943SRS017247\_contig\_number\_9909SRS017433\_contig\_number\_13086SRS017521\_contig\_number\_30892SRS017622\_contig\_number\_contig-100\_833.834SRS017821\_contig\_number\_31156SRS018133\_contig\_number\_8726SRS018623\_contig\_number\_contig-100\_235.129371SRS018817\_contig\_number\_17863SRS018836\_contig\_number\_25796SRS018888\_contig\_number\_9949SRS018984\_contig\_number\_21155SRS019030\_contig\_number\_12976SRS019068\_contig\_number\_41414SRS019161\_contig\_number\_17564SRS019397\_contig\_number\_contig-100\_387.80811SRS019445\_contig\_number\_contig-100\_122.200477SRS019601\_contig\_number\_27783SRS020271\_contig\_number\_contig-100\_907.24671SRS020328\_contig\_number\_32810SRS020394\_contig\_number\_16609SRS021219\_contig\_number\_12027SRS021484\_contig\_number\_15280SRS022609\_contig\_number\_42381SRS023526\_contig\_number\_28611SRS024075\_contig\_number\_13504SRS024132\_contig\_number\_653SRS024435\_contig\_number\_40523SRS024492\_contig\_number\_12847SRS024625\_contig\_number\_3527SRS043701\_contig\_number\_2300SRS043768\_contig\_number\_16550SRS043841\_contig\_number\_11247SRS044535\_contig\_number\_15843SRS045713\_contig\_number\_4051SRS046369\_contig\_number\_3879SRS046717\_contig\_number\_7781SRS047014\_contig\_number\_6810SRS047044\_contig\_number\_contig-100\_2772.83495SRS047433\_contig\_number\_14109SRS048164\_contig\_number\_28260SRS048870\_contig\_number\_10895SRS049402\_contig\_number\_17158SRS049446\_contig\_number\_12438SRS049773\_contig\_number\_19615SRS049773\_contig\_number\_36961SRS049995\_contig\_number\_33836SRS050026\_contig\_number\_7095SRS050925\_contig\_number\_30989SRS051031\_contig\_number\_23546SRS051610\_contig\_number\_8539SRS051882\_contig\_number\_12889SRS052697\_contig\_number\_19321SRS053214\_contig\_number\_3569SRS053398\_contig\_number\_20128SRS054352\_contig\_number\_15237SRS054905\_contig\_number\_21069SRS055017\_contig\_number\_18135SRS058770\_contig\_number\_4862SRS062654\_contig\_number\_25071SRS063040\_contig\_number\_36607SRS063190\_contig\_number\_14390SRS063370\_contig\_number\_10508SRS063518\_contig\_number\_19849SRS063985\_contig\_number\_contig-100\_92.162495SRS064757\_contig\_number\_24559SRS065504\_contig\_number\_29422SRS065504\_contig\_number\_33432SRS075773\_contig\_number\_23493SRS076804\_contig\_number\_20234SRS077086\_contig\_number\_10792SRS077194\_contig\_number\_12579SRS077231\_contig\_number\_18943SRS077294\_contig\_number\_16401SRS077552\_contig\_number\_12981SRS077641\_contig\_number\_13129SRS077849\_contig\_number\_17968SRS078176\_contig\_number\_13972SRS078242\_contig\_number\_28464SRS078419\_contig\_number\_31236SRS078665\_contig\_number\_9089SRS097958\_contig\_number\_13631SRS098571\_contig\_number\_47659SRS098644\_contig\_number\_31800SRS098827\_contig\_number\_3438SRS098881\_contig\_number\_18540SRS100021\_contig\_number\_7934SRS101376\_contig\_number\_15564SRS103987\_contig\_number\_20985SRS104036\_contig\_number\_44SRS1041118\_contig\_number\_11225SRS104311\_contig\_number\_1594SRS104636\_contig\_number\_30935SRS104975\_contig\_number\_7912SRS105153\_contig\_number\_16972SRS1054716\_contig\_number\_contig-100\_1048.40177SRS140492\_contig\_number\_33911SRS140513\_contig\_number\_15758SRS142503\_contig\_number\_29448SRS142890\_contig\_number\_22414SRS143417\_contig\_number\_24822SRS143598\_contig\_number\_contig-100\_996.136233SRS143876\_contig\_number\_contig-100\_57.232205SRS143991\_contig\_number\_16893SRS144183\_contig\_number\_36182SRS144362\_contig\_number\_45789SRS144506\_contig\_number\_19084SRS146764\_contig\_number\_4996SRS147139\_contig\_number\_4091SRS147271\_contig\_number\_31635SRS147346\_contig\_number\_66174SRS147557\_contig\_number\_31374SRS148091\_contig\_number\_4203SRS148196\_contig\_number\_17992SRS148424\_contig\_number\_30710SRS148721\_contig\_number\_47699SRS148784\_contig\_number\_14410SRS149181\_contig\_number\_28815SRS149325\_contig\_number\_11233SRS892882\_contig\_number\_contig-100\_206.42444SRS893252\_contig\_number\_14949SRS893279\_contig\_number\_4437SRS893341\_contig\_number\_contig-100\_5655.65967
140
28118
species


SRS043001\_contig\_number\_9650SRS098514\_contig\_number\_contig-100\_42.3917
species
1897010
2

2
1263090
species

SRS019381\_contig\_number\_19699SRS056259\_contig\_number\_20612

family
815
1

1
genus
816


SRS077730\_contig\_number\_6201
1
species
28116
